# Targeted peripheral focused ultrasound stimulation attenuates obesity-induced metabolic and inflammatory dysfunctions

**DOI:** 10.1038/s41598-021-84330-6

**Published:** 2021-03-03

**Authors:** Tomás S. Huerta, Alex Devarajan, Tea Tsaava, Arvind Rishi, Victoria Cotero, Christopher Puleo, Jeffrey Ashe, Thomas R. Coleman, Eric H. Chang, Kevin J. Tracey, Sangeeta S. Chavan

**Affiliations:** 1 Institute of Bioelectronic Medicine, The Feinstein Institutes for Medical Research, Northwell Health, 350 Community Drive, Manhasset, NY 11030 USA; 2 Donald and Barbara Zucker School of Medicine at Hofstra/Northwell, 500 Hofstra Blvd, Hempstead, NY 11549 USA; 3 Department of Pathology and Laboratory Medicine, Donald and Barbara Zucker School of Medicine at Hofstra/Northwell, Hempstead, NY USA; 4 GE Research, 1 Research Circle, Niskayuna, NY 12309 USA; 5 The Elmezzi Graduate School of Molecular Medicine, 350 Community Drive, Manhasset, NY 11030 USA

**Keywords:** Biological techniques, Physiology

## Abstract

Obesity, a growing health concern, is associated with an increased risk of morbidity and mortality. Chronic low-grade inflammation is implicated in obesity-driven metabolic complications. Peripheral focused ultrasound stimulation (pFUS) is an emerging non-invasive technology that modulates inflammation. Here, we reasoned that focused ultrasound stimulation of the liver may alleviate obesity-related inflammation and other comorbidities. After 8 weeks on a high-fat high-carbohydrate “Western” diet, C57BL/6J mice were subjected to either sham stimulation or focused ultrasound stimulation at the porta hepatis. Daily liver-focused ultrasound stimulation for 8 weeks significantly decreased body weight, circulating lipids and mitigated dysregulation of adipokines. In addition, liver-focused ultrasound stimulation significantly reduced hepatic cytokine levels and leukocyte infiltration. Our findings demonstrate the efficacy of hepatic focused ultrasound for alleviating obesity and obesity-associated complications in mice. These findings suggest a previously unrecognized potential of hepatic focused ultrasound as a possible novel noninvasive approach in the context of obesity.

## Introduction

More than 650 million adults worldwide have obesity, defined as a body mass index (BMI) of 30 or above ^1^. Obesity is associated with increased mortality and a number of comorbidities, such as insulin resistance, metabolic syndrome, cardiovascular disease, high cholesterol, type 2 diabetes mellitus, coronary artery disease, and nonalcoholic fatty liver disease ^2–4^. Together, these complications result in a net global economic impact of $2.0 trillion ^5^. During the period of 1980–2013, the proportion of overweight adults increased by 28% in developed countries and 60% in developing countries, with no country reporting a decrease in prevalence during the same period ^6^.

Despite an urgent need for a treatment for obesity, no specific long-lasting therapeutic approaches are currently available. Although weight loss through increased physical activity and dietary alterations can be beneficial for alleviating obesity and its adverse consequences, these lifestyle modifications are difficult to implement and sustain ^7^. While bariatric surgery can be effective for some individuals, it is associated with significant risks and is currently recommended only for individuals with morbid obesity ^8^. Finally, despite robust attempts from multiple companies, safe and effective pharmacological approaches to treat obesity remain elusive ^9^.

Preclinical and clinical studies implicate chronic low-grade inflammation in the pathophysiology of obesity, evidenced by increased circulating levels of proinflammatory cytokines, acute-phase proteins such as C-reactive protein (CRP) and altered levels of adipokines (such as adiponectin and leptin) ^10^. Excessive adipose tissue releases cytokines, chemokines and adipokines, which are associated with hyperglycemia, insulin resistance, dyslipidemia and hypertension ^11, 12^. Increased adipose mass is correlated with autonomic nervous system dysfunction, including increased sympathetic activity, reduced vagus nerve activity and sympatho-vagal imbalance ^13^. Decreased vagus nerve activity has been implicated in the development of hemodynamic and metabolic dysfunction in obesity ^14, 15^. Vagus nerve regulation of hepatic glucose production is decreased in high fat diet-induced obesity ^16^, and stimulation of cholinergic signaling pathways pharmacologically or through vagus nerve stimulation (VNS) suppresses appetite and reduces body weight ^17, 18^. Clinical studies using implanted nerve stimulators indicate VNS can induce weight reduction in obese patients ^19^. Together, these studies suggest that peripheral inflammation and peripheral nervous system are involved in the metabolic dysfunctions associated with obesity.

The current use of VNS requires surgical intervention to implant a neuromodulating device directly on the cervical vagus nerve. Recently, however, noninvasive focused ultrasound has been used for neuromodulation in both central and peripheral nervous systems to preferentially excite or inhibit neurons, with low risks ^20–23^. We recently used targeted peripheral focused ultrasound stimulation (pFUS) directed at the porta hepatis to alter glucose metabolism in endotoxemia, and demonstrated that selective activation of specific target sites modulated specific anti-inflammatory and metabolic effects ^24, 25^. Accordingly, here we reasoned pFUS of the porta hepatis, may alleviate obesity-related inflammation and other complications.

## Results

### Hepatic pFUS lowers body weight gain, food intake, and adiposity in Western diet-fed mice

**Figure 1:**
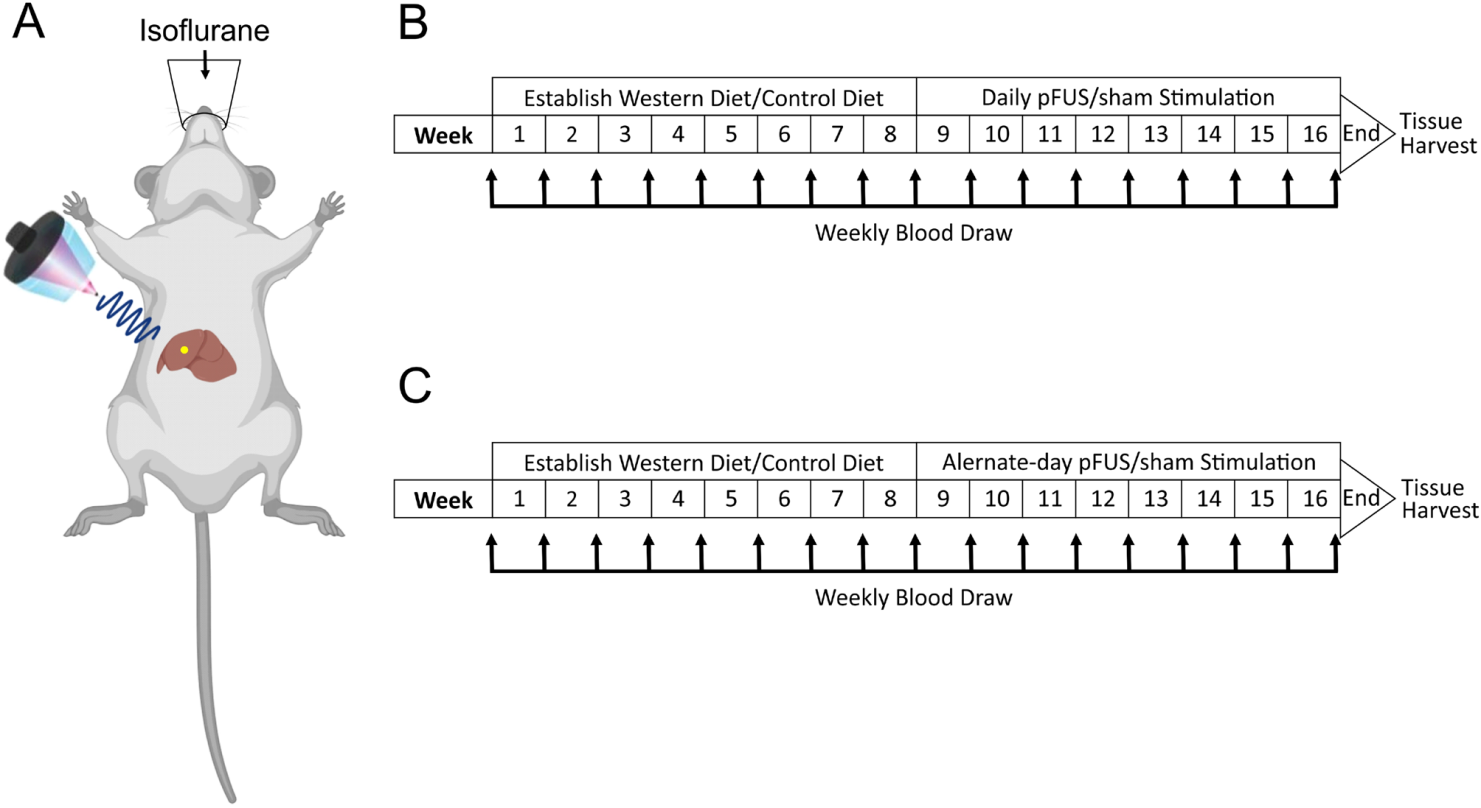
Experimental set up of peripheral focused ultrasound stimulation (pFUS). (**A**) Schematic showing pFUS targeting of the liver created using BioRender (https://biorender.com/icon/species/rodents/mouse-supine-with-organs/). Anesthetized mice are laid on the supine position, while the conical focused ultrasound transducer is aimed at the porta hepatis. Region of stimulation is represented by the yellow circle. (**B**) Experimental timeline for daily stimulation. Mice are given 8 weeks to establish either the Western diet or the control diet, then are subjected to hepatic pFUS from weeks 9–16, with weekly blood draws. At the end of week 16, mice are euthanized and tissues are harvested. **C.** Experimental timeline for alternate-day stimulation. The alternate-day stimulation paradigm is identical to the daily stimulation, except mice receive stimulation every other day for weeks 9–16.

**Figure 2:**
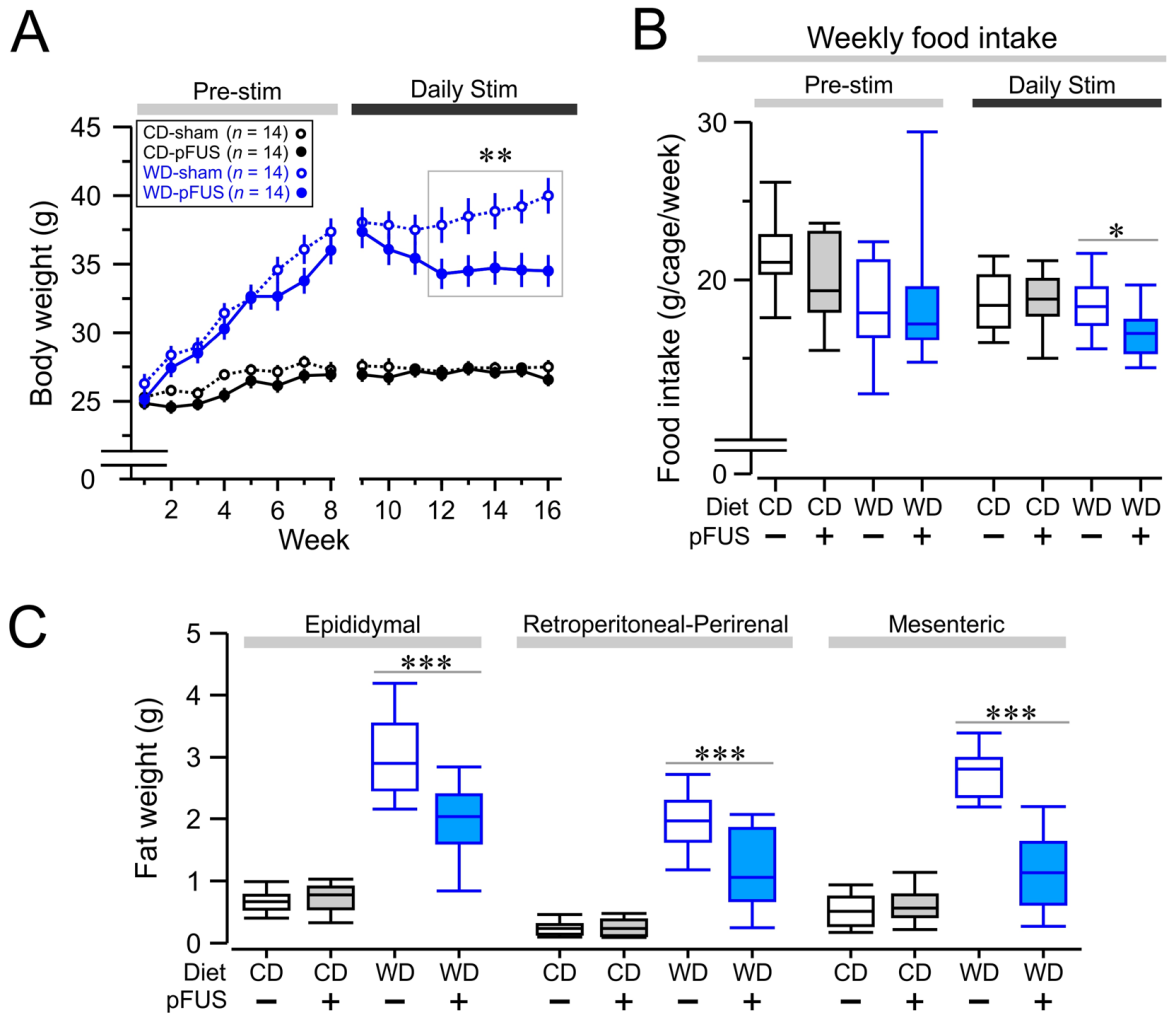
Hepatic pFUS reduces body weight gain, food intake, and abdominal adiposity in Western diet-fed mice. (**A**) Body weight as a function of time under the indicated conditions. Mice were given either Western diet (WD, blue plots) or control diet (CD, black plots) for a period of 8 weeks. On week 9, mice either received daily peripheral focused ultrasound stimulation (pFUS; closed circles, solid line) or sham stimulation (open circles, dashed line) for the remainder of the experiment. Starting at week 12, the Western diet–pFUS group (closed blue circles) had significantly attenuated body weight in comparison to Western diet–sham controls (** *P* < 0.01,  two-way RMANOVA, week 12 WD–pFUS vs WD–sham). (**B**) Western diet-fed mice have reduced food intake after ultrasound stimulation. The food intake of the mice was monitored and calculated per cage per week. No significant difference was found among any of the groups in the pre-stimulation period (*P* > 0.05,  one-way ANOVA, weeks 1–8). Post-stimulation food intake was monitored, and the food intake of the Western diet–pFUS group was found to be significantly reduced (* *P* < 0.05, two-way ANOVA, WD–pFUS vs WD–sham). **C.** Hepatic pFUS reduces abdominal adiposity. Fat weight (g) was measured on three visceral fat pads (Epidydimal, Retroperitoneal/Perirenal, and Mesenteric) extracted postmortem. The Western diet–pFUS group had significantly lower fat weight in the three fat pads compared to the Western diet–sham group (Epidydimal & Mesenteric, *** *P* < 0.001, two-way ANOVA; Retroperitoneal/Peririenal, **** *P* < 0.0001, two-way ANOVA).

To assess the therapeutic efficacy of hepatic pFUS for obesity, animals were fed either high-fat (60% kcal from fat) high-carbohydrate (55% fructose and 45% glucose supplement in water) “Western diet” or low-fat (10% kcal from fat, calorie matched to the high-fat diet) low carbohydrate (normal water) “control diet” for 8 weeks prior to stimulation (Fig. 1B). The mice on the Western diet gradually increased weight over the course of the first 8 weeks, which reached a difference of ~ 10 g when compared to mice fed a low-fat control diet (Fig. 2A, *P* < 0.001, *F*_(3,39)_ = 49.93,  two-way RM ANOVA). Beginning at week 9, mice on both diets were randomized into two subgroups that received either daily sham stimulation or pFUS targeted to the porta hepatis for the remainder of the study, until week 16 (Fig. 1A,B). This produced 4 distinct groups: control diet–sham stimulation, control diet–pFUS, Western diet–sham stimulation, and Western diet–pFUS.

The mice on Western diet subjected to sham stimulation continued to gain weight from weeks 9–16. Hepatic pFUS gradually attenuated the body weight gain, reaching a significant difference with the sham stimulated group by week 12 (Fig. 2A, *P* < 0.01, *F*_(3,39)_ = 49.93, two-way RMANOVA). No significant difference was observed between the control diet-fed groups that received either pFUS or sham stimulation (Fig. 2A). Average weekly food intake was calculated for the pre-stim period (week 1–8) and post-stim period (9–16) as the difference between the filled chow and end-of-week chow weights, per mice in each cage, per number of weeks. The average weekly food intake, which did not differ significantly between the four groups prior to the stimulation period, was reduced in the Western diet group subjected to pFUS as compared to the sham group (Fig. 2B, *P* < 0.05, *F*_(3)_ = 6.27, one-way ANOVA). The abdominal adiposity of the mice was assessed post-mortem by harvesting three sites of fat tissue (epididymal, retroperitoneal/perirenal, and mesenteric). Mice on the Western diet that received sham stimulation had significantly increased fat tissue weights in all three of the fat deposits as compared to mice on the control diet (both sham and pFUS groups) (Fig. 2C), and hepatic pFUS significantly reduced abdominal adiposity as compared to sham stimulated group in Western diet group (Fig. 2C, *P* < 0.01, *F*_(3,33)_ = 127.6, two-way RMANOVA). Together these results indicate hepatic pFUS decreases body weight gain, reduces food intake, and moderates abdominal fat pad accumulation in Western diet-fed obese mice.

### Hepatic pFUS attenuates dysregulation of adipokines in Western diet-fed mice

**Figure 3:**
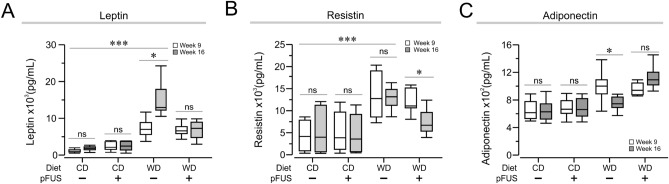
Hepatic pFUS attenuates dysregulation of adipokines in Western diet-fed mice. (**A**) Western diet-sham mice have increased leptin compared to control diet-sham mice (*** *P* < 0.001, two-way ANOVA). pFUS attenuates leptin increase seen in Western diet–sham mice (WD–sham, week 9 vs week 16, ** *P* < 0.01, two-way ANOVA). (**B**) Western diet-sham mice have increased resistin compared to control diet-sham mice (*** *P* < 0.001, two-way ANOVA). pFUS reduces resistin levels in Western diet–pFUS group (* *P* < 0.05, two-way ANOVA). (**C**) pFUS attenuates the reduction in adiponectin levels seen in Western diet–sham mice (WD–sham, week 9 vs week 16, * *P* < 0.05, two-way ANOVA).

Serum collected at week 9 and week 16 were assessed for leptin, MCP-1, PAI-1, resistin, TNF, IL-1β, IL-6, glucagon, GLP-1, C-peptide, and ghrelin. Leptin and resistin levels were significantly elevated in the Western diet-fed mice at week 9 as compared to mice on control diet (Fig. 3A, week 9 leptin, *P* = 0.001, *F*_(2)_ = 30.3, one-way ANOVA; Fig. 3B, week 9 resistin, *P* = 0.001, *F*_(2)_ = 13.41_,_ one-way ANOVA). Further, leptin levels continue to increase from week 9 to week 16 in Western diet group subjected to sham stimulation, whereas hepatic pFUS attenuated the increase (Fig. 3A, *P* = 0.05, *F*_(2)_ = 58.39, two-way ANOVA). Resistin levels remained elevated in Western diet-fed sham stimulated mice between week 9 and 16, whereas pFUS lead to a significant decrease in resistin levels at week 16 as compared to week 9 (Fig. 3B, *P* < 0.05, *F*_(2)_ = 25.05, two-way ANOVA). Decreased adiponectin levels in obesity inversely correlate with obesity-associated complications ^26^. Plasma adiponectin levels were significantly lower in Western diet-fed sham stimulated mice at week 16 as compared to week 9 (Fig. 3C, *P* < 0.05, *F*_(2)_ = 27.64, two-way ANOVA). Hepatic pFUS treatment prevented the decrease of adiponectin levels seen in Western diet-fed mice given sham stimulation (Fig. 3C). No differences were observed in the serum levels of MCP-1, PAI-1, TNF, IL-1β, IL-6, glucagon, GLP-1, C-peptide, or ghrelin.

### Hepatic pFUS reduces circulating lipid levels in Western diet fed mice

**Figure 4:**
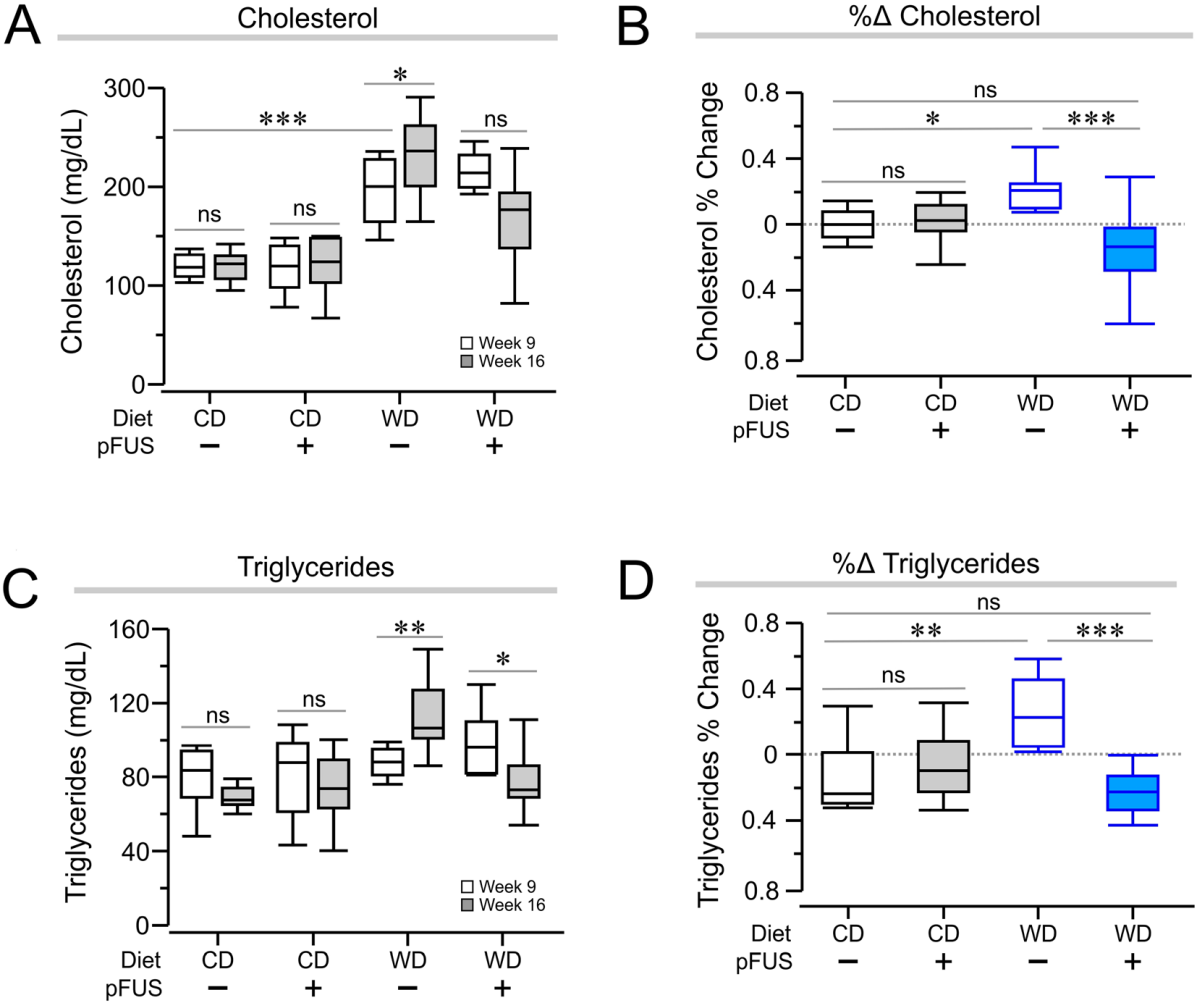
Hepatic pFUS attenuates levels of circulating lipids. (**A**) At week 9, Western diet–sham and Western diet–pFUS mice had significantly increased cholesterol levels when compared to the control diet–pFUS group. (*** *P* < 0.001, two-way ANOVA). Hepatic pFUS attenuates total cholesterol increase seen in Western diet–sham mice (WD–sham, week 9 vs week 16, * *P* < 0.05, two-way ANOVA). (**B**) Percent change of cholesterol levels between weeks 9 and 16 reveal that the Western diet–pFUS group has a percent decrease that is significantly different from the Western diet–sham group (*** *P* < 0.001, one-way ANOVA) (**C**) Hepatic pFUS lowers circulating triglyceride levels shown to increase in Western diet–sham mice (WD–sham, week 9 vs week 16, ** *P* < 0.01; WD–pFUS, week 9 vs week 16, * *P* < 0.05, two-way ANOVA). (**D**) Percent change of triglyceride levels between weeks 9 and 16 demonstrate that the Western diet–pFUS percent decrease is significantly different from the Western diet–sham percent increase (*** *P* < 0.001, one-way ANOVA).

We observed significantly higher plasma total cholesterol levels in mice on Western diet as compared to control diet at week 9 (Fig. 4A, week 9 cholesterol, *P* < 0.001, *F*_(2)_ = 31.06, one-way ANOVA), which continued to increase in sham stimulated group. This increase was attenuated by hepatic pFUS (Fig. 4A, *P* < 0.05, *F*_(2)_ = 52.67, 2-way ANOVA). Similarly, plasma triglyceride levels significantly increased between weeks 9 and 16 in the Western diet group subjected to sham stimulation (Fig. 4C, *P* < 0.01, *F*_(2)_ = 13.66, two-way ANOVA) and hepatic pFUS reduced these levels significantly (Fig. 4C, *P* < 0.05, *F*_(2)_ = 13.66, two-way ANOVA). Thus, hepatic pFUS significantly attenuated the increase in both cholesterol and triglyceride levels observed between weeks 9 and 16 in Western diet-fed mice (Fig. 4B, *P* < 0.001, *F*_(2)_ = 9.85, Fig. 4D, *P* < 0.001, *F*_(2)_ = 13.14, one-way ANOVA).

### Hepatic pFUS attenuates alanine aminotransferase levels, liver weight, proinflammatory cytokine and hepatic leukocyte infiltration in Western diet-fed mice

**Figure 5:**
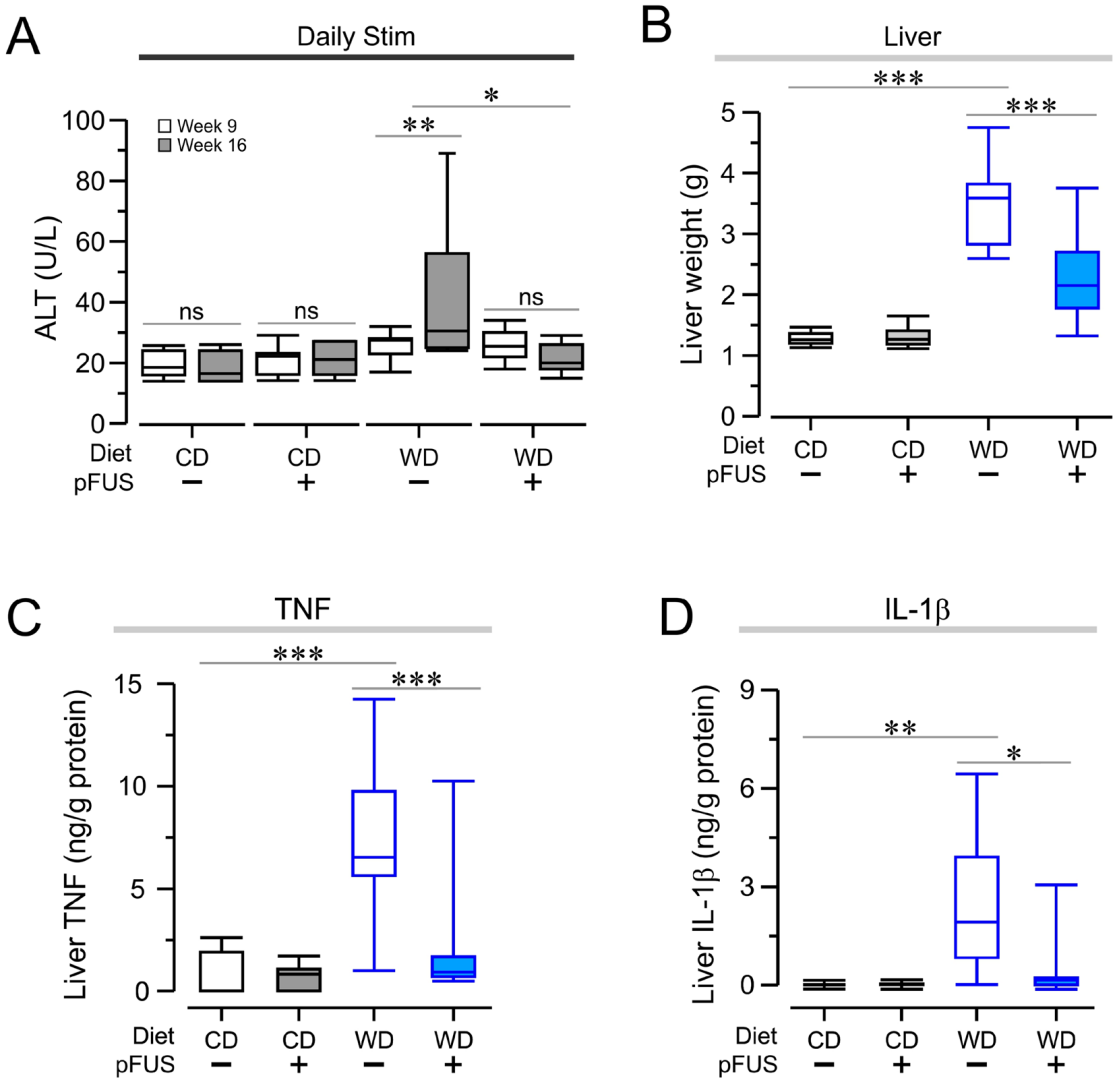
Hepatic pFUS attenuates circulating ALT, reduces gross liver weight, and diminishes proinflammatory cytokines levels. (**A**) pFUS attenuates ALT increase seen in Western diet–sham mice (WD–sham, week 9 vs week 16, ** *P* < 0.01, two-way ANOVA). pFUS treatment reduces endpoint ALT levels (* *P* < 0.05, two-way ANOVA). (**B**) Hepatic pFUS reduces liver weight. Western diet–sham mice had significantly increased liver weight compared to control diet–sham mice (*** *P* < 0.001, one-way ANOVA). Western diet–pFUS mice had significantly lower liver weight compared to Western diet–sham mice (*** *P* < 0.001, one-way ANOVA). (**C,D**) Hepatic pFUS lowers proinflammatory cytokines in the liver. Wester diet–sham mice had increased TNF levels compared to control diet–sham mice (**C**, *** P < 0.001, one-way ANOVA). Hepatic pFUS lowered TNF levels in Western diet-fed mice (**C**, *** *P* < 0.001, one-way ANOVA). Western diet–sham mice had increased IL-1β levels compared to control diet–sham mice (**D**, ** P < 0.01, one-way ANOVA). Hepatic pFUS stimulated Western diet-fed animals had lower IL-1β levels (**D**, * *P* < 0.05, one-way ANOVA).

**Figure 6:**
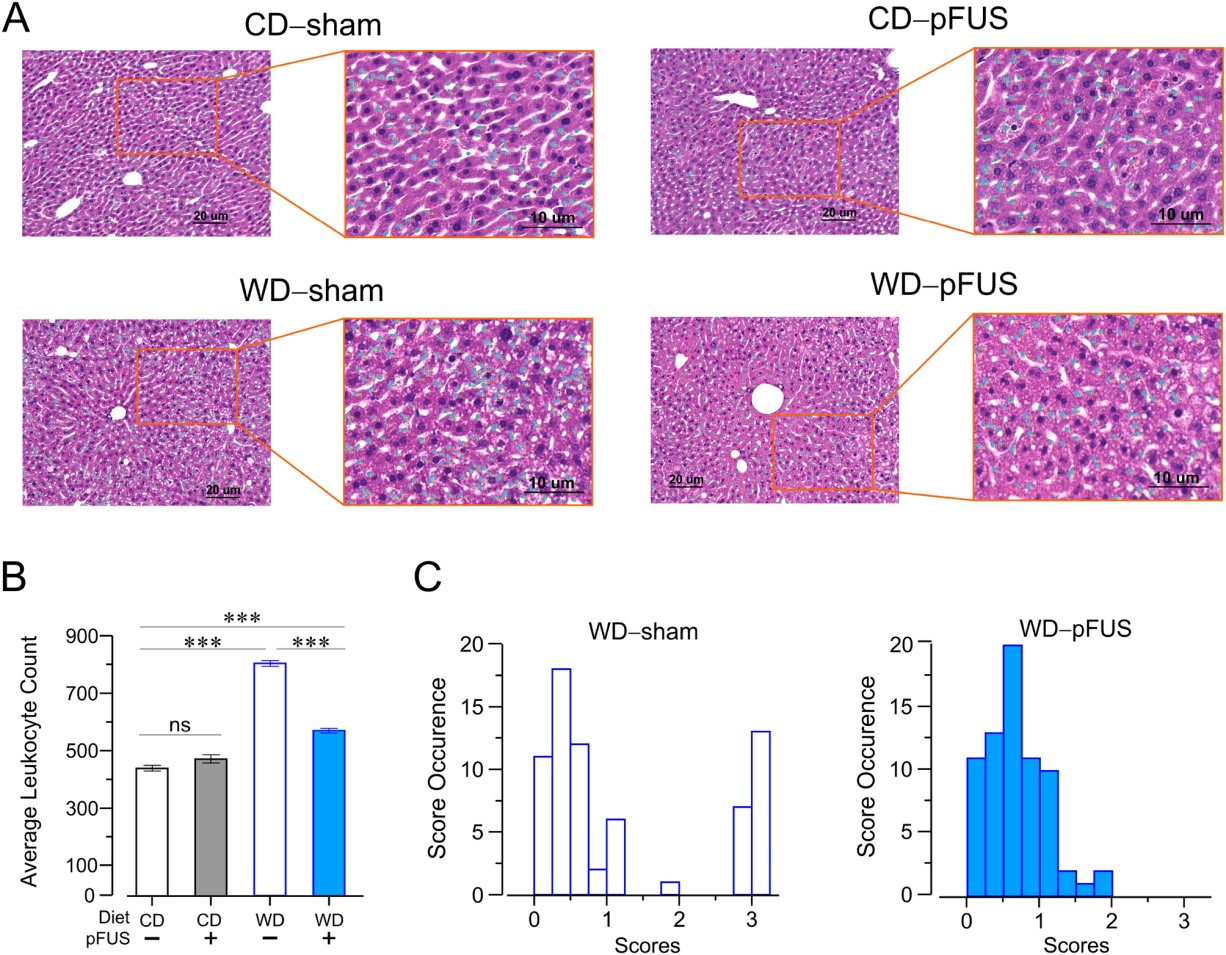
pFUS reduces the volume and severity of leukocyte infiltration in Western diet-fed mice. (**A**) Representative images of H&E stained sections with semi-automated mask for counting immune cells. (**B**) Semi-automatic counting of infiltrating leukocytes. Western diet–pFUS mice had reduced the average number of leukocytes counted per slide compared to Western diet–sham mice (WD–sham, n = 134 sides; WD–pFUS, n = 134 slides; *** *P* < 0.001, one-way ANOVA). The control diet–sham group had significantly lower counts than the Western diet–pFUS group (CD–sham, n = 64 slides; WD–pFUS, n = 134 slides; *** *P* < 0.001, one-way ANOVA). (**C**) Severity of immune cell infiltration was quantified and scored in a blinded fashion by a pathologist. The Western diet–sham group had a higher score for inflammation (score 3 is severe), compared to the Western diet–pFUS group (*P* < 0.01, Kolmogorov–Smirnov test).

At week 16, alanine aminotransferase (ALT) levels were significantly increased in Western diet-fed mice subjected to sham stimulation (Fig. 5A, *p* < 0.01, *F*_(2)_ = 6.12, two-way ANOVA), and hepatic pFUS significantly alleviated ALT levels (Fig. 5A, *P* < 0.05, *F*_(2)_ = 6.12, two-way ANOVA). These alterations were consistent with the changes observed in liver weights in sham stimulated Western diet-fed mice as compared to control diet-fed mice (Fig. 5B, *P* < 0.001, *F*_(3)_ = 52.84, one-way ANOVA), and a significant liver weight decrease following hepatic pFUS (Fig. 5B, *P* < 0.001, *F*_(3)_ = 52.84, one-way ANOVA). Analysis of hepatic cytokine levels revealed a significant increase in proinflammatory cytokines, TNF and interleukin-1β (IL-1β), in Western diet-fed mice as compared to control diet-fed mice (Fig. 5C, TNF, *P* < 0.001, *F*_(3)_ = 14.37, one-way ANOVA; Fig. 5D, IL-1β, *P* < 0.01, *F*_(3)_ = 7.25, one-way ANOVA). Hepatic pFUS significantly reduced TNF and IL-1β levels in the Western diet-fed mice (Fig. 5C, *P* < 0.001, *F*_(3)_ = 14.37, one-way ANOVA; Fig. 5D, *P* < 0.05, *F*_(3)_ = 7.25, one-way ANOVA). Liver tissue sections were stained with hematoxylin and eosin (H&E) for histological assessment for the severity of steatohepatitis and leukocyte infiltration (Fig. 6A). pFUS was not found to cause a significant change in the severity of steatohepatitis in Western diet-fed mice. A significantly higher leukocyte count in livers of Western diet-fed mice was observed as compared to the control diet-fed mice (Fig. 6B, *P* < 0.0001, Kruskal–Wallis H test). Hepatic pFUS significantly reduced leukocyte count in the livers of Western diet-fed mice as compared to sham stimulated mice (Fig. 6B, *P* < 0.0001, Kruskal–Wallis H test). Analysis of the severity of the inflammation in a blinded manner revealed a reduction in the leukocyte infiltration following hepatic pFUS as compared to sham stimulated controls (Fig. 6C, *P* < 0.01, Kolmogorov–Smirnov D test). Together, these data demonstrate that hepatic pFUS reduces ALT levels, liver weight, proinflammatory cytokines and leukocyte infiltration in the livers of Western diet-fed mice.

### Hepatic pFUS induces a dose-dependent effect of on weight gain and obesity-related metabolic sequalae

**Figure 7:**
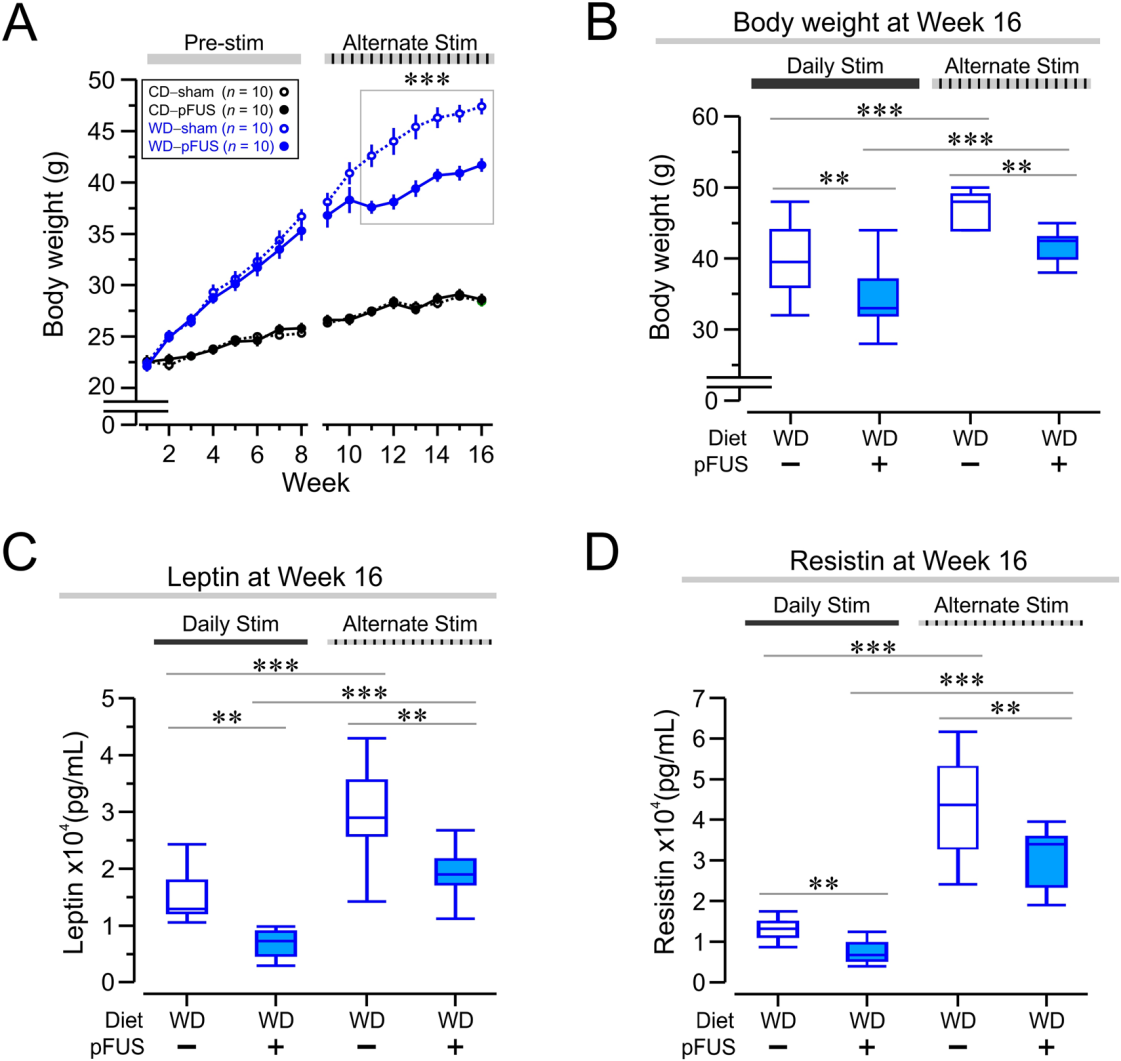
Dose-dependent effects of pFUS on weight gain and obesity-related metabolic derangements. (**A**) Alternate-day pFUS reduces the severity of body weight gain in Western diet-fed mice. By week 11, the Western diet–pFUS group (closed blue circles) had significantly lower body weights than the Western diet–sham group (open blue circles; *** *P* < 0.001, two-way RMANOVA). The sham groups did not differ significantly from each other throughout the study. (**B**) Alternate-day hepatic pFUS reduces body weight gain, but less robustly than daily stimulation. The endpoint weights of the daily stimulated Western diet-fed groups were compared to the alternate-day stimulated Western diet-fed groups. The daily stimulated Western diet–pFUS group was significantly lower than the alternate-day stimulated group (*** *P* < 0.001, two-way ANOVA, daily WD–pFUS vs alternate WD–pFUS). (**C**) Alternate-day stimulation attenuates leptin hormone elevation in Western diet–pFUS mice. At week 16, both daily and alternate-day stimulation regimens were sufficient to significantly lower leptin levels (** *P* < 0.01, two-way ANOVA). Comparison of daily and alternate-day stimulated Western diet–pFUS groups revealed that daily ultrasound stimulation significantly decreased leptin compared to alternate-day stimulation (*** *P* < 0.001, two-way ANOVA) (**D**) Hepatic pFUS significantly reduced resistin levels in both daily stim and alternate-day Western diet–pFUS groups compared to their respective Western diet–sham groups (** *P* < 0.01, two-way ANOVA). The daily stimulated Western diet–pFUS group had significantly lower resistin compared to alternate-day Western diet–pFUS (*** *P* < 0.001, two-way ANOVA).

To provide insight into the dose-dependent effect of hepatic pFUS, we subjected separate groups of mice to either control diet or Western diet for 8 weeks (Fig. 1C), followed by hepatic pFUS on alternate days instead of daily. As anticipated, the Western diet-fed mice increased in body weight between weeks 1–8, which by week 9 reached a difference of ~ 12 g as compared with the body weight of control diet-fed mice (Fig. 7A, week 9, *P* < 0.0001, *F*_(3)_ = 173.4, two-way RMANOVA). At week 9, the animals were divided into subgroups that received either sham stimulation or hepatic pFUS on alternate days until week 16. Alternate-day hepatic pFUS in Western diet-fed mice reduced the rate of body weight gain significantly by week 11 as compared to sham stimulated controls (Fig. 7A, *P* < 0.001, *F*_(3)_ = 173.4, two-way RM ANOVA). This body weight difference persisted until the end of the experiment. Comparison of the body weight at week 16 across daily stimulation and alternate-day stimulation groups showed that daily stimulation was more effective at lowering body weight than alternate-day stimulation (Fig. 7B, *P* < 0.001, *F*_(3)_ = 22.61, one-way ANOVA). Comparison between the Western diet-fed sham-stimulated groups revealed that the alternate-day stimulation mice had higher body weights than their daily stimulated counterparts (Fig. 7B, *P* < 0.001, *F*_(3)_ = 22.61, one-way ANOVA), suggesting a dose-dependent effect of hepatic pFUS.

The adipokines leptin and resistin were significantly decreased in Western diet-fed mice subjected to hepatic pFUS at week 16 as compared to their sham stimulated controls in both the daily and alternate-day stimulation paradigms (Fig. 7C, leptin, *P* < 0.01, *F*_(3)_ = 26.55, one-way ANOVA; Fig. 7D, resistin, *P* < 0.01, *F*_(3)_ = 49.87, one-way ANOVA). Comparison across the daily and alternate-day stimulated Western diet-fed mice for the week 16 time point revealed increased leptin and resistin levels in alternate-day stimulated group as compared to the daily stimulated mice (Fig. 7C, leptin daily vs. alternate-day, *P* < 0.001, *F*_(3)_ = 26.55, one-way ANOVA; Fig. 7D, resistin daily vs. alternate-day, *P* < 0.001, *F*_(3)_ = 49.87, one-way ANOVA). The difference in the sham-stimulated groups demonstrates the robust effect of exposure to anesthesia on metabolic function, during daily or alternate-day stimulation. Together, these data indicate that there is a dose-dependent effect of hepatic pFUS on weight gain and obesity-related metabolic derangements in Western diet-fed mice.

## Discussion

Here we report that noninvasive focused ultrasound targeted to the liver porta hepatis significantly ameliorates the severity of obesity and obesity-associated inflammatory and metabolic derangements in mice on a high-calorie, high-fat Western diet. Hepatic pFUS significantly reduces body weight, food intake, and abdominal adiposity, attenuates inflammatory cytokines and circulating lipids, alleviates dysregulation of adipokines, and improves liver pathology in obese mice. Using an alternate-day stimulation schedule, we observed a dose-dependent effect of hepatic pFUS for reducing body weight and adipokines. These results indicate a previously unrecognized application of focused ultrasound targeted at the porta hepatis for alleviating obesity and obesity-associated complications.

A major contributing factor to the obesity epidemic is the consumption of high-calorie, high-fat foods ^27^. Accordingly, we utilized a well-established Western diet-induced model of obesity in C57BL/6J mice ^28–30^. A number of studies have highlighted the use of focused ultrasound as a novel noninvasive methodology to stimulate nerves with a sizeable window of safe stimulation, prior to exhibiting any heat effects or tissue damage ^22, 23, 31, 32^. Focused ultrasound is known to have varying degrees of efficacy depending on the stimulation parameters ^24, 25^. Here, we utilized a previously discovered set of stimulation parameters optimized for end-organ stimulation in rodents ^24^.

The observed reduction in body weight and abdominal fat accumulation can be partially attributed to the food intake suppressing effect of hepatic pFUS. Peripheral signals from many organs including white adipose tissue, gut, pancreas and liver are known to regulate body weight and food intake ^33–37^. Despite its well-established role in the control of food intake, the liver has not been a target for the development of therapeutic strategies to modulate appetite and treat obesity. Here, we show for the first time that direct focused ultrasound stimulation of the porta hepatis, a major neural access route to the liver ^33, 34^, moderates food intake and weight gain, consistent with a neuromodulatory component mediating the therapeutic effects of hepatic pFUS. These results are in line with a previous study that showed focused ultrasound has no measurable effect in healthy rats ^24.^ Hepatic pFUS did not change feeding behavior of mice on control diet, suggesting a selective effect during metabolic dysregulation. The regulation of feeding behavior and appetite by the liver is multifaceted, and a vital role for cholinergic signaling through the vagus nerve has been previously reported ^38^. The vagus nerve serves as a primary conduit between the liver and the central nervous system ^39^. In both preclinical and clinical settings, VNS has been shown to reduce body weight ^40–42^. Hepatic vagal afferents in the porta hepatis are uniquely positioned to respond rapidly to nutrients following intestinal absorption. Changes in neural activity following hepatic pFUS may, therefore, alter the response of the vagus nerve to the nutrients ^33, 43, 44^. Indeed, hepatic branch vagotomies induce increased food intake and body weight in rodent models, demonstrating a requirement for vagus nerve signaling in this process ^34^. Significantly, hepatic vagus fibers are mainly located in the porta hepatis and not in the liver lobules ^33, 34^. Collectively, these findings raise the possibility that specifically targeting the porta hepatis to activate hepatic vagus activity may provide new targets for the development of therapeutic strategies for appetite control and obesity.

The levels of adipocyte-derived hormones, leptin, resistin and adiponectin, depend on the fat mass and are involved in the endocrine, immunological and metabolic complications of obesity. A common finding in obesity is chronically elevated leptin and resistin levels and reduced adiponectin levels, and association of adipokine dysregulation with inflammation ^26, 45^. Low-grade chronic inflammation, with excess fat driving the release of proinflammatory cytokines such as TNF and IL-1β  ^46^, is associated with adiposity, insulin resistance, hyperleptinemia, metabolic syndrome and type 2 diabetes ^47–51^*.* Although the serum levels of proinflammatory cytokines were below detection limits, we observed a significant reduction in the hepatic levels of proinflammatory cytokines TNF and IL-1β, indicating a selective anti-inflammatory effect of the focused ultrasound in the stimulated end-organ. It is possible that through alleviating the dysregulation of adipokines and inflammatory cytokines (TNF and IL-1β) in obese conditions by hepatic focused ultrasound stimulation may, in turn, mediate beneficial effects on obesity-associated complications including food intake, leptin resistance and liver pathophysiology.

Abdominal adiposity and weight gain have been proposed as the main driving forces of inflammation and insulin resistance in obesity ^52^. Decreasing visceral fat and body weight could be contributing factors to attenuating inflammatory state in Western diet-fed mice exposed to pFUS. However, while hepatic pFUS treatment reduced hepatic inflammatory mediators to levels detected in control diet-fed mice, pFUS exposed obese mice were still significantly heavier and with higher visceral adiposity than the control diet-fed mice. These results suggest a specific anti-inflammatory effect of pFUS that cannot be attributed simply to reduced body weight and abdominal adiposity. We have recently shown that splenic pFUS significantly attenuates endotoxin-induced inflammation in preclinical models. We have also demonstrated that hepatic pFUS-induced modulation of glucose homeostasis is centrally mediated, and may activate afferent vagus signaling ^24^, suggesting that hepatic pFUS-mediated modulation of inflammatory responses via neural signaling through afferent pathways. Continued work is needed to further explore pFUS-induced changes in afferent vagus nerve signaling using electrophysiological recordings.

An aspect of pFUS treatment that is unexplored is the optimal treatment frequency for pFUS delivery. For example, pFUS in rodents is largely done on a daily basis ^53^, while human treatment ranges from a single stimulation to three times per week ^54^. In order to address whether frequency of hepatic ultrasound stimulation treatment has a dose-dependent effect on the therapeutic outcome in obesity, we subjected a second cohort of mice to hepatic pFUS on an alternate-day schedule (Fig. 7). While alternate day pFUS was effective for improving some metabolic functions, daily pFUS presented a more robust therapeutic effect in several metabolic measures. Collectively, these results suggest that the efficacy of pFUS treatment in a chronic setting of obesity has a dose dependence to the regularity of treatment, with more frequent pFUS administration being more effective in this case.

The observations made in this study have several limitations. Notably, the mechanism of action of focused ultrasound is not fully understood. Cavitation (generation of small bubbles within the cell) and membrane deformation induce neuronal activation in peripheral nerves by disrupting receptors on the cell membrane ^55^. Conversely, thermal effects due to ultrasound stimulation suppress neuronal activity ^56^. Ultrasound stimulation may also directly activate mechanosensitive channels such as the transient receptor potential (TRP) channel family and Nav1.8 channels, which are expressed on the vagus nerve innervating the porta hepatis. One or several of these mechanisms of action may be relevant to our experiments, which warrants further investigation. Furthermore, the focal point of the ultrasound stimulation in mice is large enough to include the liver tissue immediately surrounding the nerves in mice. Thus, the ultrasound effect may also be attributed to an indirect effect due to stimulation of the surrounding hepatocytes or immune cells. Further, hepatic pFUS may be utilized in future experiments to assess the degree of steatosis, ballooning, fibrosis and possible steatohepatitis in obese mice, as these liver dysregulations are key comorbidities to obesity. Finally, the ultrasound effect, although sufficient to reduce obesity and improve several aspects of metabolic health, was not sufficient to completely abolish the body weight gain seen in Western diet-fed mice. These results warrant a study where mice on the Western diet are later switched to a control diet to determine if hepatic focused ultrasound can fully alleviate the symptoms of obesity.

Collectively, our findings demonstrate the efficacy of end-organ targeted focused ultrasound to the porta hepatis for alleviating obesity and improving several aspects of metabolic health in mice. As the prevalence and societal impact of diet-induced obesity continues to rise, safe and noninvasive approaches to treat metabolic impairments are urgently needed. Although future work addressing the mechanisms are warranted, our results highlight the exciting possibility of using hepatic focused ultrasound as one such novel noninvasive treatment for obesity with numerous potential clinical applications.

## Methods

### Animals

Experiments were performed on male C57BL/6J mice (8 weeks old, Jackson Lab, Bar Harbor, ME, USA). All procedures performed in compliance with the ARRIVE guidelines and in accordance with the National Institutes of Health (NIH) Guidelines under protocols approved by the Institutional Animal Care and Use Committee (IACUC) of the Feinstein Institutes for Medical Research, Northwell Health.

### Experimental design

6–8 week old C57BL/6J mice were fed regular chow for 10 d in a reverse light cycle room, and then switched to a high-fat diet (D12492, 60% kcal from fat), or its corresponding isocaloric low-fat diet (10% kcal from fat) for 16 weeks. Mice were group-housed 5 per cage, with cage density matched between groups. Food and bedding were changed weekly, and the weekly food consumption per cage was measured as the difference between the filled chow weight and the end-of-week chow weight. Any chow pellets that fell through the grating were included in the end-of-week measurement, however smaller food particulates were changed with the bedding. Mice in the high-fat Western diet group received sugar supplemented water (55% fructose, 45% sucrose). After 8 weeks, the Western diet-fed mice were divided into two groups, either treated with pFUS of the porta hepatis (once daily) or sham stimulation for the following 8 weeks. After 8 weeks, the low-fat control diet mice were treated with either the pFUS or the sham stimulation for the remaining 8 weeks (represented in Fig. 1B). The sample size of this experiment was n = 15 per group (60 total). A second cohort of mice underwent the same dietary regiment, but only received alternate-day hepatic pFUS during the stimulation period (week 9–16, Fig. 1C). The sample size of the second cohort was n = 10 per group (40 total). Body weights for all the mice (n = 100) were monitored on a weekly basis. No exclusion criteria was applied for this experiment, and the study was determined to have a large effect size (Cohen’s *d* = 1.194199, confidence interval 95%). At the end of the experiment, mice were euthanized and liver weight, visceral adipose weight, cytokine and adipokine levels, metabolic profile, liver histology were evaluated.

### Peripheral focused ultrasound stimulation

Mice that received pFUS were anesthetized at 2% isoflurane at 1 L/min O_2_, for the stimulation period (5 min). Mice were then placed in the supine position on a water circulating warming pad, with a rectal thermometer probe to maintain body temperature. The area above the stimulation target was shaved and hair was fully removed with Nair. The porta hepatis was localized using a custom ultrasound imaging device (GE Research) ^24^. The location was marked with a permanent marker and a focused ultrasound stimulation probe (GE Research) was placed on the target area (represented in Fig. 1A). The device then delivered 1 min of stimulation (1.1 MHz, 200 mV per pulse, 150 burst cycles, 500 μs burst period), followed by a 30 s period of rest, then a subsequent 1 min of stimulation ^24^. Sham mice had their hair removed, and went under anesthesia for 5 min with the pFUS probe placed on the marked target area, similarly to the pFUS group.

### Blood collection and tissue harvesting

After a morning fast (3–4 h) blood was collected at week 9 and week 16 using the cheek bleed method. Approximately 300 µL of whole blood was sampled per animal. Blood samples were spun in a centrifuge (10 min at 5000 rpm, then 2 min at 10,000 rpm) and the serum was extracted and frozen for further evaluation.

At the end of the study, mice were subjected to an overnight fast. After body weight measurement and blood collection via cheek bleed, mice were euthanized by CO_2_ asphyxiation. Mice were perfused with 4% PFA. Three sites of previously defined fat tissue epididymal, retroperitoneal/perirenal, and mesenteric were harvested, and weighed ^57^. Livers were excised, rinsed with saline and weighed. The largest lobe of the liver was sectioned for H&E staining.

### Serum adipokine determination and other blood biochemistry tests

Serum samples were centrifuged from whole blood drawn by cheek bleeding (10 min at 5000 rpm, then 2 min at 10,000 rpm). The samples were then analyzed with a Millipore MILLIPLEX mouse adipokine panel assay for leptin, MCP-1, PAI-1, resistin, TNF, IL-1β, IL-6, glucagon, GLP-1, C-peptide, and ghrelin. Serum samples were assessed with a Piccolo Xpress chemistry analyzer (Abbott Laboratories, Abbott Park, IL) using a Lipid Panel Plus: cholesterol, HDL, triglycerides, ALT, AST, nHDLc, total cholesterol/HDL, LDL, and VLDL. Serum adiponectin was measured by using a Mouse Adiponectin ELISA (Invitrogen, Carlsbad, CA, USA) according to manufacturer’s recommendations.

### Liver histology and hepatic inflammation assessment

Livers were fixed by a perfusion of 4% PFA, and then the largest lobe of the liver was embedded in paraffin. The lobe was then sliced, and the liver tissue sections were subjected to hematoxylin and eosin (H&E) staining. Glass slides were then prepared from the formalin fixed, paraffin embedded tissue. Steatosis and steatohepatitis were graded a hepatobiliary pathologist with experience of looking at clinical liver biopsies. Pathological evaluation was blinded to the experimental groupings. Inflammation was evaluated as the number of foci (cluster n ≥ 5) of inflammatory cells, either lymphocytes or neutrophils. Inflammation was assessed in 5 different fields at 20 × magnification and the average was scored between 0–3: score 0 = normal, no inflammatory foci, score 1 = slight, 1–2 foci/20 × field, score 2 = moderate, 3–4 foci/20 × field, and score 3 = severe, > 4 foci/20 × field.

### Quantification of inflammatory cells in the liver

H&E stained liver sections were analyzed for cell count using a Keyence BZ-X800 microscope. Images of the liver sections were acquired at 20 × magnification and the Hybrid Cell Count function was used to threshold, filter, and count the number of leukocytes in the field-of-view. The parameters for the cell count were optimized to avoid counting of nuclei, while counting as many leukocytes as possible. The upper limit for cell area was 30 µm^2^, aperture stop was 100%, transmitted light was set to 50%, and brightness (exposure) was set to 1/100 s. The same parameters were used to batch process all liver images.

### Statistical analysis

Data were analyzed using GraphPad Prism 7 software. Data in X–Y plots are expressed as mean ± SEM. Significant differences between normal data sets were assessed by using either one way analysis of variance (ANOVA), or 2-way ANOVA where appropriate. Longitudinal data was assessed using a repeated measures (RM) two way ANOVA (RMANOVA), with post-hoc multiple comparisons analysis. Significant differences between nonparametric datasets were assessed by Kruskal–Wallis H test and Kolmogorov–Smirnov D test. Effect size was calculated for endpoint body weights by Cohen’s *d* test at 95% Confidence Interval. Differences with *P* < 0.05 were considered statistically significant.
